# Multi-layer resource configuration and safety optimization for integrated modular avionics with resource sharing and isolation

**DOI:** 10.1371/journal.pone.0345130

**Published:** 2026-03-31

**Authors:** Hongli Wang, Zhiwei Chen, Xiaotong Fang

**Affiliations:** 1 School of Safety Engineering, China University of Labor Relations, Beijing, China; 2 Unmanned system research institute, Northwestern Polytechnical University, Xi’an, Shanxi, China; 3 China Institute of Ship Integrated Technology and Economics, Beijing, China; Universitas Mercatorum, ITALY

## Abstract

The aviation industry extensively employs integrated modular avionics (IMA) to enhance system efficiency by sharing resources across various functions. Despite the benefits, the design of IMA systems is not without its challenges, particularly in achieving cost-effectiveness, ensuring availability, and addressing safety concerns. Optimizing resource utilization in IMA systems is increasingly complex due to the growing functionality and quantity of resources in aviation modules and technological flexibility. This paper presents a multi-layer resource configuration and optimal design method for IMA systems, with a dual focus on resource sharing and isolation mechanisms (which prevent fault propagation and enhance system safety). Our approach takes into account not only the strategic planning of resource sharing but also the intricacies of isolation scheme design, especially in the context of risk propagation. The architecture of the multi-layer resource configuration is meticulously structured and formalized to account for the unique dynamics of risk propagation across different configurations. The optimization process for the configuration scheme is formalized as a constrained multi-objective optimization problem. An optimal performance configuration scheme for the IMA systems can be identified that requires the minimum amount of resources. Finally, the effectiveness of the proposed method is demonstrated through the presentation of an illustrative example. The results show that the proposed approach effectively balances safety, efficiency, and cost in IMA resource management.

## 1. Introduction

Integrated modular avionics (IMA) system is a platform that provides a shared set of flexible, reusable, and interoperable hardware and software resources. This platform is meticulously designed and rigorously verified to fulfill a defined set of safety and performance requirements for hosting aircraft functions [1], such as reliability, real-time performance, partition protection, and fault tolerance as specified in standards like DO-178C [2] and DO-254 [3]. The IMA architecture utilizes a sharing mechanism through modular and universal resources, as opposed to the traditional dedicated resources [4,5]. By enabling the sharing of multiple resources, the IMA architecture not only improves efficiency but also leads to savings in terms of space, weight, power, and maintenance infrastructure [6].

However, the sharing mechanism, while enhancing the utilization efficiency of resources, can also potentially lead to fault propagation and diffusion [7–9]. As the extent of resource sharing increases, the risk of fault coupling propagation and diffusion escalates alongside the improvement in resource utilization efficiency. To mitigate this risk, an isolation mechanism (also referred to as isolation design) is implemented to bolster the system safety of integrated modular avionics, ensuring that failures in one partition do not affect others. For instance, different application software is encapsulated within distinct partitions, employing specific isolation strategies within partition management. These partitions are effectively isolated from each other, thereby significantly enhancing the system’s fault tolerance capability [10]. The efficiency, safety, and availability of the system are contingent upon the effective configuration of resources for both shared and isolated designs. Achieving the optimal configuration that strikes a balance between resource sharing and isolation, ensuring system safety while maintaining high availability and efficiency, remains a significant challenge. Currently, although certain research has addressed aspects of this issue, there remains a gap in methods for optimizing IMA configuration.

Currently, research on IMA systems primarily emphasizes system architecture and hardware integration levels, with limited exploration of resource configuration within the IMA architecture. To systematically position our work, we categorize and review existing literature along three interlinked dimensions: (1) resource configuration modeling, (2) safety-integrated design, and (3) multi-objective optimization. **1) Resource Configuration Modeling**: The foundation of IMA design lies in effectively mapping functions to shared hardware resources. Early work by Sagaspe et al. [11] pioneered a safety analysis method that considers the impact of resource sharing, employing model checking to derive safety constraints for configuration generation. Building on this, Sagaspe [12] proposed a constraint-based design approach that incorporates isolation requirements to determine system implementability on an IMA platform. To tackle the combinatorial complexity of mapping. Lohse [13] developed an optimization algorithm that generates and estimates new system variables, while Salzwedel [14] focused on optimizing resource configuration during the early system design phase to enhance architectural robustness. **2) Safety-Integrated Design and Scheduling**: Ensuring safety within shared-resource environments necessitates integrating safety constraints into scheduling and allocation. Annighöfer [15,16] addressed this by developing scheduling tools that resolve time-division issues and practical constraints using integer programming for multi-objective optimization (e.g., weight, cost). From a verification perspective [17], modeled and verified software/hardware resource configurations to ensure real-time performance and safety. Similarly, Cai [18] proposed an adaptive multi-objective optimization algorithm that simultaneously considers access time, resource type, quantity, and isolation constraints. For systems with stringent reliability needs, Xie et al. [19] optimized resource schemes by balancing hardware cost, real-time requirements, and reliability, and Chu [20] formalized the configuration optimization for systems with functional redundancy as a hybrid constraint satisfaction and optimization problem. **3) Multi-Objective Optimization for Modern Constraints**: Recent research has expanded objectives to include energy efficiency and communication scheduling. Chen et al. [21,22] developed energy-constrained scheduling algorithms for heterogeneous systems. Du et al. [23] advanced this direction by employing an improved ant colony optimization algorithm for energy-aware resource allocation. In the realm of platform configuration, Moraes [24] integrated Monte Carlo tree search with genetic algorithms to explore trade-offs in platform design. To address scheduling inflexibility, Zhou et al. [25] introduced a loosely coupled partition-message scheduling framework that decouples task and message scheduling, reducing complexity imposed by tight coupling, **Research Gap and Our Contribution:** While the aforementioned studies provide valuable insights, two critical gaps remain. First, most works adopt an economic or schedulability-centric perspective; safety is often treated as a binary constraint rather than a quantifiable objective interacting dynamically with resource sharing and isolation mechanisms. The modeling of risk propagation pathways and their impact on system safety under different configuration schemes is notably underdeveloped. Second, there is a lack of a unified, multi-layer resource configuration model that holistically optimizes the trade-off between safety (e.g., quantified failure probability, real-time performance), resource utilization efficiency, and cost. Existing approaches often focus on a single layer or a subset of objectives, failing to capture the hierarchical interdependencies in IMA systems where configuration decisions at one layer (e.g., partition) affect risk and performance at others.

Therefore, this paper addresses the following research question: How can we optimize the multi-layer resource configuration in IMA systems to systematically balance resource sharing (for efficiency) and isolation (for safety), while explicitly quantifying and mitigating the risk of fault propagation? Targeted at the aforementioned challenges and constraints, and taking the inherent trade-off between resource sharing and isolation into account, this paper proposes a method to optimize the design of multi-layer resource configuration in an IMA system. Our approach aims to mitigate the risk of fault association propagation due to resource sharing and to strike a balance between sharing and isolation, thereby attaining a safe and efficient optimal configuration.

To achieve this, high real-time performance, high reliability, and efficiency—which are crucial metrics for safety-critical complex embedded systems—are formally utilized as conditions for safety optimization. They are encapsulated as constraints within our comprehensive assessment framework to ensure the viability of all candidate configuration schemes. Specifically, this paper introduces a shared resource configuration optimization model alongside a comprehensive optimization process. The effectiveness of this process is illustrated through the application of a formal mathematical model and the Non-Dominated Sorting Genetic Algorithm-II (NSGA-II), showcasing its successful implementation in a representative case study. The proposed method provides technical support for achieving efficient, safe, and balanced resource configuration optimization for IMA systems.

The remainder of this paper is organized as follows. Section 2 introduces the IMA system and its shared and isolation design for resources. Section 3 proposes a configuration model for IMA resource sharing and isolation. Section 4 describes the configuration optimization process. In Section 5, we demonstrate the applicability of the proposed technique through a case study of the Integrated Display and Control System (IDCS). Finally, Section 6 concludes the paper by summarizing the research paths that deserve more attention from the community in the near future and drawing concluding remarks.

## 2. IMA system, resource sharing and isolation design

### 2.1. IMA system description

**Fig 1:**
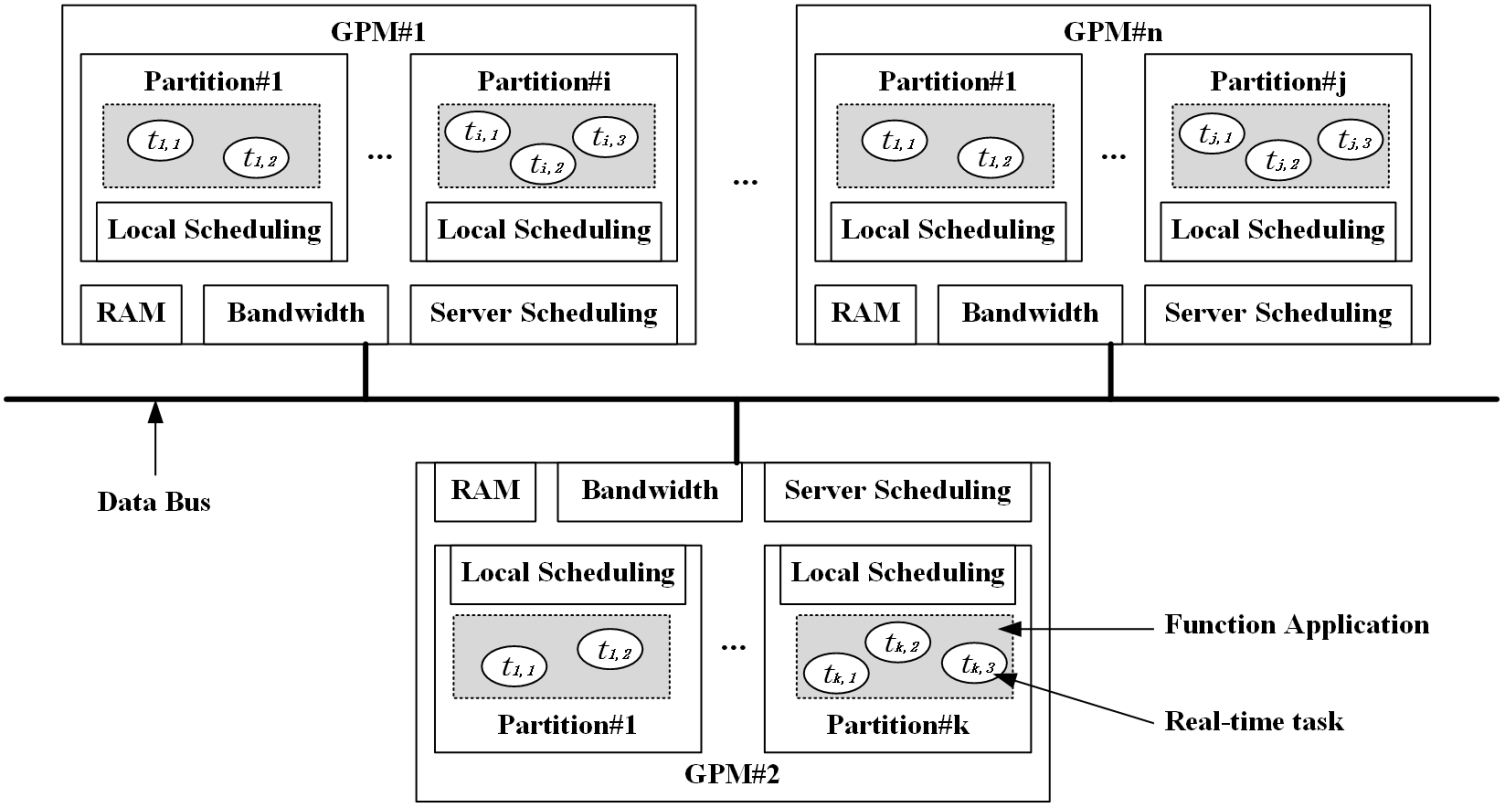
Overview of IMA resource sharing and isolation mechanism.

An IMA system’s core consists of a cluster of entities encompassing numerous shared processing resources capable of simultaneously supporting functions across different missions [4]. As illustrated in Fig 1, these resources are interconnected by data buses, constituting a distributed network system known as the shared platform. Each function is deployed independently within a partition. The real-time tasks associated with a specific function application are performed within the respective partition through a local scheduling mechanism. Furthermore, a central server scheduling mechanism oversees the management of all partitions across a processing resource. To accommodate a variety of target tasks, a pre-designed configuration scheme dictates the deployment of these functions within the shared platform.

**Fig 2:**
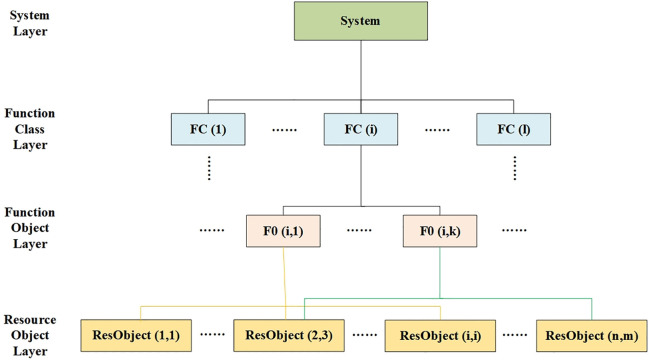
System-function-resource layered mapping model for IMA.

The IMA system can be conceptualized as comprising resources and Hosted Functions (HFs). To fulfill its designated tasks, a single HF may require support from multiple resources. The interconnections between different HFs are depicted in Fig 2. Within the IMA system, there are various classes of HFs, each serving distinct purposes, with multiple function objects potentially existing beneath each class. The function objects within a particular class may establish backup relationships with each other for safety reasons or work collaboratively to improve task execution.

As shown in Fig 2, the IMA architecture follows a layered mapping: the system level comprises multiple hosted functions (HFs), each of which is implemented by a set of resources. This mapping forms the basis for configuring sharing and isolation. The model consists of three layers: 1) System Layer: Represents the overall IMA platform. 2) Function Layer (Hosted Functions, HFs): Each HF class (e.g., Navigation, Communication) contains multiple function objects that may collaborate or provide redundancy. 3) Resource Layer: Physical or logical resources (e.g., GPMs, partitions) that implement the functions. Arrows indicate allocation relationships: a function may require multiple resources, and resources may be shared across functions.

However, while the design of tasks and functions operates at the logical layer of the system, their execution relies on the utilization of physical resources. Specifically, the management of each function involves the collaboration of multiple resources to establish an allocation mapping from the logical to the physical resource layer.

**Fig 3:**
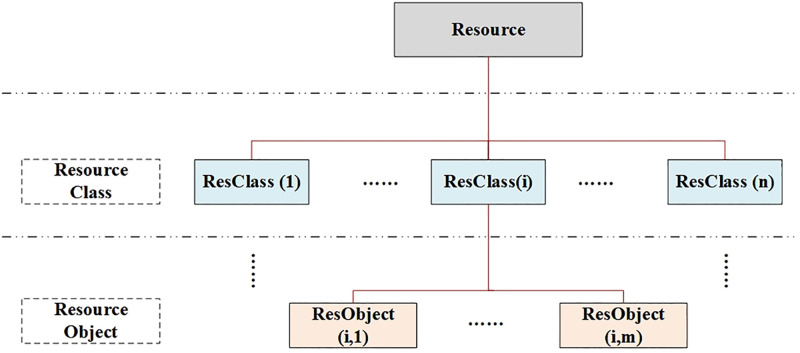
Resource class-object hierarchy and allocation relations.

Similarly, Fig 3 illustrates how resources are structured into classes and objects. This hierarchy supports flexible allocation and enables both sharing (multiple functions using the same resource object) and isolation (dedicated resource objects for safety-critical functions). Resources are organized into classes (e.g., General Processing Module (GPM), memory, bus) and instances (e.g., GPM1, GPM2). 1) Class level: Defines resource type and properties. 2) Object level: Specific instances that can host multiple function objects. Dashed lines represent potential sharing relationships; solid lines indicate allocation. Each resource objects can accommodate multiple function objects.

### 2.2. Shared resources and isolation design

The shared resources in an IMA system include physical shared resources (e.g., processors, memory) and data shared resources (e.g., databases, algorithms). Physical sharing is realized through time- and space-division multiplexing, while data sharing enables reuse and result exchange across functions. Both forms improve resource utilization but introduce potential fault propagation risks. To mitigate fault propagation risks introduced by sharing, isolation mechanisms (e.g., partitioning, dedicated resources) are employed. In this work, isolation refers to the design strategies that prevent fault propagation between functions or resources. Isolation can be categorized as: 1) Temporal Isolation: Functions are allocated non-overlapping time slots on shared resources (e.g., via time-partitioning in ARINC 653). 2) Spatial Isolation: Functions are assigned to physically separate resources (e.g., dedicated processors or memory regions). 3) Logical Isolation: Functions are separated through software mechanisms such as memory protection units (MPUs) or virtualization. 4) Fault-Containment Isolation: Resources are designed to limit the impact of a fault to a predefined boundary (e.g., using health monitoring and reset mechanisms). Isolation prevents low-criticality functions from affecting high-criticality ones, enhancing system safety and fault tolerance..

To clarify the concepts of resource sharing and isolation, consider an IMA system with two functions: 1) Function A: Flight Control (high criticality); 2) Function B: Data Logging (low criticality).

**Shared Resource Design**: Both functions may be allocated to the same General Processing Module (GPM) using time partitions. This improves resource utilization but introduces a risk: a fault in Function B could propagate to Function A via the shared GPM.

**Isolated Resource Design**: Function A is assigned a dedicated GPM, while Function B uses a separate GPM. This eliminates coupling faults but increases resource usage.

The challenge in IMA configuration is to balance these two approaches to achieve **efficienc**y (through sharing) and **safety** (through isolation), which is the focus of our optimization method.

## 3. Configuration model of IMA shared resource and isolation

To establish an efficient IMA system, it is crucial to tackle the configuration of IMA resources proactively. This approach enables the seamless integration of aircraft-level functions into the IMA system, provided that the resources are properly configured. According to the DO-297 [1] specification, the IMA platform is composed of a variety of configurable resources. It is imperative to configure these resources efficiently to facilitate the execution of diverse aircraft-level functions on the IMA platform.

### 3.1. Multi-level resource configuration

**Notation and Definitions**

For clarity, the following notation is used throughout this paper:

***S***_***R***_: set of resource elements
***n***: number of resource types
***N***_***t***_: number of resources of type *t*
$\overset{n}{\underset{N_{t}}{r}}$: the ***N***_***t***_-th resource object of type *n*
***f***_***m***_: the *m*-th upper-layer function or resource
$\overset{rf}{\underset{ij}{M}}$: resource-to-function mapping matrix, where $\overset{rf}{\underset{ij}{M}}=\text{1}$ if resource ***r***_***i***_ is allocated to function ***f***_***j***_, otherwise 0
${\left(\overset{n}{\underset{ij}{C}}\right)}_{m\times m}$: association matrix for resources of type *n*, where $\overset{n}{\underset{ij}{C}}=\text{1}$ indicates a coupling between $\overset{n}{\underset{i}{r}}$ and $\overset{n}{\underset{j}{r}}$
$\overset{n}{\underset{ij}{P}}$: probability of failure propagation from $\overset{n}{\underset{i}{r}}$ to $\overset{n}{\underset{j}{r}}$
$\overset{n}{\underset{m}{P}}$: failure probability of resource $\overset{n}{\underset{m}{r}}$, considering both inherent and propagated failures

The IMA system encompasses various resource types, including hardware, software, data, and network resources. The objective of resource configuration is to assign each resource to an appropriate location for efficient utilization. However, resource configuration must adhere to certain constraints. For example, the configuration rules prohibit assigning two different resource types to the same location, and high-criticality resources are designated as non-shareable.

Expanding on the shared resources and isolation discussed in Section 2, this section proposes a multi-layer resource configuration method for the IMA system’s resource layer. The association between software and hardware is achieved through various resource types, including node, partition, process, and variable resources. These associations are determined based on the attributes of each resource type.

The resource layer element set ***S***_***R***_ is a physical or logical unit used to enable system functions. The ***S***_***R***_ comprises all resources in the IMA system, organized by type and object. Let there be *T* types of resources, indexed by *t* = 1,2,…,T. Each resource type *t* contains ***N***_***t***_ objects. The set can be expressed as [26]:


$$S_{R}=\{(\overset{\text{1}}{\underset{\text{1}}{r}},\overset{\text{1}}{\underset{\text{2}}{r}},\overset{\text{1}}{\underset{\text{3}}{r}},\ldots ,\;\overset{\text{1}}{\underset{N_{\text{1}}}{r}}),(\overset{\text{2}}{\underset{\text{1}}{r}},\overset{\text{2}}{\underset{\text{2}}{r}},\overset{\text{2}}{\underset{\text{3}}{r}},\ldots ,\;\overset{\text{2}}{\underset{N_{\text{2}}}{r}}),\ldots ,(\overset{n}{\underset{\text{1}}{r}},\overset{n}{\underset{\text{2}}{r}},\overset{n}{\underset{\text{3}}{r}},\ldots ,\;\overset{n}{\underset{N_{t}}{r}})\}$$ (1)

in which, $\overset{n}{\underset{N_{t}}{r}}$ denotes the ***N***_***t***_-th object of resource type ***n***, n∈(1,2,…,N)**,** $N_{t}\in (\text{1},\text{2},\ldots ,T)$. ***N***_***t***_ is the total number of objects of type *t*. The first superscript indicates the resource type, the second indicates the object index. For example, $\overset{\text{3}}{\underset{\text{2}}{r}}$ represents the second resource object allocated from the third type of resource.

Once all shared resources have been allocated according to a specific configuration scheme, a multi-layer resource configuration can be established. At this time, the coupling association relationship within the resource layer can be determined. An adjacency matrix can be used to describe this relationship as a formal representation. Specifically, the mapping association configuration matrix between resource elements (***r***_***n***_) and upper-layer resources (***f***_***m***_) can be expressed as:


$$\begin{array}{c} M^{rf}={\overset{rf}{\underset{ij}{(M}})}_{n\times m}=\begin{array}{cc} \begin{array}{c} \begin{array}{c} \;\\ r_{\text{1}} \end{array}\\ \begin{array}{c} r_{\text{2}}\\ \begin{array}{c} \vdots \\ r_{n} \end{array} \end{array} \end{array} & \begin{array}{c} \begin{array}{cc} f_{\text{1}} & \;f_{\text{2}} \end{array}\;\begin{array}{cc} \ldots & \;f_{m} \end{array}\\ \left[\begin{array}{ccc} \begin{array}{cc} \overset{rf}{\underset{\text{1}\text{1}}{M}} & \overset{rf}{\underset{\text{1}\text{2}}{M}}\\ \overset{rf}{\underset{\text{2}\text{1}}{M}} & \overset{rf}{\underset{\text{2}\text{2}}{M}} \end{array} & \cdots & \begin{array}{c} \overset{rf}{\underset{\text{1}m}{M}}\\ \overset{rf}{\underset{\text{2}m}{M}} \end{array}\\ \vdots & \ddots & \vdots \\ \begin{array}{cc} \overset{rf}{\underset{n\text{1}}{M}} & \overset{rf}{\underset{n\text{2}}{M}} \end{array} & \cdots & \overset{rf}{\underset{nm}{M}} \end{array}\right] \end{array} \end{array} \end{array}$$ (2)

The allocation matrix $M^{rf}$, where a column sum of 1 indicates exclusive allocation (spatial isolation). where, the shared resource configuration in the upper layer resource is 1. If the shared resource is not allocated to the upper-layer resource, it is 0. So $\overset{rf}{\underset{ij}{M}}$ can be expressed as:


$$\overset{rf}{\underset{ij}{M}}=\left\{ \begin{array}{c} \text{1},\text{ Shared resource }r_{i}\text{ is allocated to }\\ \text{functional}/\text{upper}-\text{level resources }f_{j}\;\\ \;\text{0},\text{Shared resource }r_{i}\text{ is not allocated to }\\ \text{functional}/\text{upper}-\text{level resources }f_{j} \end{array}\right.\;$$ (3)

Since the bottom resources of complex integrated system support the realization of the upper functions, the configuration of resources should be based on the functional requirements. ${HF}^{l}$ denotes Class ***l*** function. Multiple sub-functions realize a class of functions $\overset{l}{\underset{k}{HF}}$, ${HF}^{l}=\left\{\overset{l}{\underset{\text{1}}{HF}},\overset{l}{\underset{\text{2}}{HF}},\cdots ,\overset{l}{\underset{k}{HF}}\right\}$. ***k*** is the number of sub-functions, k=(1,2,…,N).

### 3.2. Safety constraint model

IMA resource sharing and isolation can be configured in various ways to satisfy the constraints. However, different configuration approaches have varying impacts on aircraft performance, installation cost, and safety. Thus, identifying the optimal resource configuration within these constraints is a critical issue. In this section, we first model the shared resource configuration. We then express the corresponding safety constraints using mathematical expressions. This approach facilitates the optimal configuration of resource sharing and isolation. The specific constraints are as follows:

**Constraint 1**. The number of each bottom-layer shared resource cannot exceed the total number of upper-layer resources.

This constraint indicates that there is at least one 1 in the allocation of $\left\{\overset{\text{1}\text{2}}{\underset{n\text{1}}{M}},\overset{\text{1}\text{2}}{\underset{n\text{2}}{M}},\cdots ,\overset{\text{1}\text{2}}{\underset{nm}{M}}\right\}$ of the lowest level resource $\overset{\text{1}}{\underset{N_{y}}{r}}$, but it is less than or equal to the sum of the upper level resources configured, $\text{1}\leq \sum_{j=\text{1}}^{m}\overset{\text{1}\text{2}}{\underset{nj}{M}}\leq m$; A set of inequalities is used to describe the constraint:


$$b_{\text{1}}\leq M^{\text{1}\text{2}}A_{\text{1}}\leq b_{m}$$ (4)

among them, $A_{\text{1}}=[\text{1},\text{1},\cdots ,\text{1}{]}^{T}$ is a summation vector, $b_{\text{1}}=[\text{1},\text{1},\cdots ,\text{1}{]}^{T}$ ensures each resource is used at least once, $b_{m}=[m,m,\cdots ,m{]}^{T}$ limits the maximum allocation to *m* functions. Example: If there are *m = 3* upper-layer functions, a shared resource can be assigned to 1, 2, or all 3 functions, but not to none or more than 3.

By multiplying the associated configuration matrix $\overset{\text{1}\text{2}}{\underset{nj}{M}}\;$ (formula (2) in formula (4)), it can be seen that the sum of the values of elements in each row of $M^{\text{1}\text{2}}$ is between 1 and m, so this constraint condition is met, every shared resource is all used.

What needs illustration is that: When $M^{\text{1}\text{2}}A_{\text{1}}=b_{\text{1}}$, the number of configurations of each resource is 1. If $M^{\text{1}\text{2}}A_{\text{1}}\to b_{\text{1}}$, the higher the degree of resource sharing is. If $M^{\text{1}\text{2}}A_{\text{1}}\to b_{m}$, the lower the degree of resource sharing is, the higher the degree of isolation design is. When $M^{\text{1}\text{2}}A_{\text{1}}=b_{m}$, each resource adopts isolated safety design, that is, all resources are not shared.

**Constraint 2**. Constraints on the number of dedicated resources.

A shared resource contains dedicated resources, which support only one upper-layer resource or sub-function. Therefore, dedicated resources cannot be shared. That is, the number of dedicated resources corresponding to ***h*** upper-layer resources or sub-functions can only be ***h***, and ***h*** ≤ ***t***, where ***t*** is the upper-layer resource or sub-function. The configuration number of this special resource constraints $\overset{n}{\underset{N_{t}}{r}}$ has to be 1, namely the values of $\left\{\overset{rf}{\underset{n\text{1}}{M}},\overset{rf}{\underset{n\text{2}}{M}},\cdots ,\overset{rf}{\underset{nm}{M}}\right\}$ have only one 1, namely $\sum_{j=\text{1}}^{m}\overset{rf}{\underset{nj}{M}}=\text{1}$. Use an equality expression to describe this constraint:


$$A_{DR(rf)}\overset{rf}{\underset{nj}{M}}=b_{DR(rf)}$$ (5)

in which $A_{DR(rf)}=[\text{1},\text{1},\cdots ,\text{1}{]}^{T}$, $b_{DR(rf)}=[\text{1},\text{1},\cdots ,\text{1}{]}^{T}$. The association configuration matrix $\overset{rf}{\underset{nj}{M}}$ represent the association configuration relationship between dedicated resources and upper-layer resources (a special case in formula (2)). By analyzing the associated configuration matrix multiplication in formula (5), it can be seen that there is only one 1 for all elements in the ***n*** row of dedicated resource $\overset{n}{\underset{N_{t}}{r}}$, and the sum of its values can only be equal to 1.

In addition, dedicated resources can be configured with other shared resources in the same upper-layer resource or sub-function. Therefore, the number of shared resources in the upper-layer resource configured with dedicated resources is ≥1, that is, $\sum_{i=\text{1}}^{n}\overset{rf}{\underset{im}{M}}\geq \text{1}$. The constraint is expressed by the inequality as:


$$A_{DR(fr)}\overset{rf}{\underset{im}{M}}\geq b_{DR(fr)}$$ (6)

in which $A_{DR(fr)}=[\text{1},\text{1},\cdots ,\text{1}]$, $b_{DR(fr)}=[\text{1},\text{1},\cdots ,\text{1}]$. For the dedicated resource $\overset{n}{\underset{N_{t}}{r}}$, all elements in the m column must have at least one 1. The sum of the values must be greater than or equal to 1.

Formula (5) states that each dedicated resource appears exactly once in the allocation matrix.

Formula (6) ensures that the upper-layer function receiving a dedicated resource also uses at least one other shared resource.

Example: A dedicated sensor $\overset{\text{1}}{\underset{\text{5}}{r}}$ is allocated only to function $f_{\text{1}}$, and $f_{\text{1}}$ also uses shared processor $\overset{\text{2}}{\underset{\text{1}}{r}}$.

**Constraint 3**. Conflicting resources cannot be allocated to the same upper-layer resource, and isolation constraints must be satisfied.

In the IMA system resource configuration process, certain resources are not allowed to be allocated to the same upper-layer resource simultaneously; these are referred to as conflicting resources. In our model, this constraint determines which resources are in conflict based on the actual situation in the configuration process. In addition, shared resources that feature isolated designs are considered conflicting and should not be allocated to the same upper-layer resource. Certain resources are designated as “conflicting”, including that: 1) Scheduling Conflicts: If two resources require simultaneous access to the same shared bus or processor, they may cause timing violations or deadline misses. 2) Access Contention: Resources that share a common physical or logical channel (e.g., memory bus, I/O port) may conflict if accessed concurrently, leading to performance degradation or data corruption. 3) Safety Isolation Requirements: For fault containment, resources of different criticality levels must be isolated. Co-allocation could allow fault propagation across safety boundaries. 4) Functional Interference: Some resources perform mutually exclusive functions (e.g., two sensors measuring the same physical quantity) and should not be assigned to the same function to avoid redundancy loss or measurement conflict. For example, suppose that the c resource object $\overset{n}{\underset{c}{r}}$ and the d resource object $\overset{n}{\underset{d}{r}}$ of the n resource are configured to the $\overset{n+\text{1}}{\underset{N_{t}}{r}}$ upper resource, that is, $\overset{rf}{\underset{cm}{M}}+\overset{rf}{\underset{dm}{M}}\leq \text{1}$. In fact, there may be multiple groups of resources that need to meet the isolation conditions. The constraint is described by an inequality:


$$A_{SEG}M^{rf}\leq b_{SEG}$$ (7)

The conflict matrix $A_{SEG}$, which enforces that conflicting resources are not co-allocated. Among them, each row in a matrix $A_{SEG}$ is assigned a value of 1 on the resource that may conflict. The number of rows in matrix $A_{SEG}$ is the number of resource groups that need to meet the isolation condition in practice, and the number of columns is the number of shared resources that are used in practice, that is, the value of n in the above. $b_{SEG}=[\begin{array}{cccc} \text{1} & \text{1} & \cdots & \text{1}\\ \text{1} & \text{1} & \cdots & \text{1}\\ \vdots & \vdots & \ddots & \vdots \\ \text{1} & \text{1} & \cdots & \text{1} \end{array}]e\times f$, the row number e of this matrix corresponds to the number of rows in $A_{SEG}$, the number of columns f corresponds to the upper resource allocation, namely the m value of the above. Each row of $A_{SEG}$ indicates a pair of conflicting resources, and $b_{SEG}$ ensures they are not co-allocated. For example: If $\overset{\text{1}}{\underset{\text{1}}{r}}$ and $\overset{\text{1}}{\underset{\text{2}}{r}}$ are conflicting (e.g., two redundant sensors that must be isolated), then $\overset{rf}{\underset{\text{1}j}{M}}+\overset{rf}{\underset{\text{2}j}{M}}\leq \text{1}$ for any function $f_{j}$.

**Constraint 4**. Safety-critical resources require a separate isolated design.

“Safety Critical Resource (SCR)” is used to describe a resource or function that has a direct impact on system safety. SCR refers to any resource or function the failure, malfunction, or absence of which could directly lead to system malfunction in a specific context. Safety critical resources or functions must be isolated and should not share the same resource, with the exception of dedicated resources, to enhance system safety. This constraint indicates that the safety-critical resource $\overset{n^{\prime }}{\underset{N_{m}}{r}}$ has only one upper-layer resource and one dedicated resource, that is, $\left\{\overset{rf^{\prime }}{\underset{\text{1}m}{M}},\overset{rf^{\prime }}{\underset{\text{2}m}{M}},\cdots ,\overset{rf^{\prime }}{\underset{nm}{M}}\right\}$ has only two 1, that is, $\sum_{i=\text{1}}^{n}\overset{rf^{\prime }}{\underset{im}{M}}=\text{2}$. Use an equality expression to describe this constraint:


$$A_{SCR}\overset{rf^{\prime }}{\underset{im}{M}}=b_{SCR}$$ (8)

among them, $A_{SCR}=[\text{1},\text{1},\cdots ,\text{1}]$, $b_{SCR}=[\text{2},\text{2},\cdots ,\text{2}]$. $\overset{rf^{\prime }}{\underset{im}{M}}$ represents the configuration relationship between the SCR and the upper-layer resource (a special case in formula (2)). By analyzing the associated configuration matrix multiplication in formula (8), it can be seen that there are two 1 for all elements in the m column where the SCR $\overset{n^{\prime }}{\underset{N_{m}}{r}}$ is located, and the sum of their values can only be equal to 2.

where $b_{SCR}=[\text{2},\text{2},\cdots ,\text{2}{]}^{\text{T}}$ ensures that the safety-critical resource and its dedicated backup are both allocated. For example: A flight control processor $\overset{\text{1}}{\underset{\text{6}}{r}}$ (safety-critical) is allocated to function $f_{\text{1}}$, along with a dedicated monitoring processor $\overset{\text{1}}{\underset{\text{5}}{r}}$.

Dedicated resources and safety-critical resources are assigned with strict isolation constraints (formula (5)–(8)).

## 4. Configuration optimization

In the previous sections, we discussed the constraints used to evaluate the effectiveness of the resource sharing and isolation configuration scheme. In this section, we propose a method to optimize the configuration scheme. Our objective is to find a resource configuration scheme that minimizes cost, ensures safety constraints, maintains real-time system performance, and achieves the highest possible resource utilization.

### 4.1. Safety formalization model

Various safety indices are employed to assess the safety of a system, including failure probability, real-time performance, and fault tolerance. Failure probability serves as a quantitative measure in safety analysis, derived from an analysis of the system’s functional structure. It sequentially infers the failure probabilities of all the system’s logical components to determine the frequency of various incidents. Real-time performance is a crucial metric for evaluating safety-critical complex embedded systems. Given the stringent requirements for high real-time performance of the IMA system, it is regarded as a safety index. In this study, failure probability and real-time performance are used as quantitative measures within the safety model to assess the safety level of shared resource and isolation configurations in the IMA system.

1) Failure probability.

In this study, safety levels are categorized into four distinct layers, and the inherent failure logic relationships are used to describe these failures, as illustrated in Fig 2. The inherent failure logic relationships among the first three layers—the system layer, the functional class layer, and the function object layer—remain consistent. Regardless of resource configuration, the inherent failure logic within the system remains unchanged.

The failure probability of the IMA system can be calculated by considering the coupling correlation failure logic relations among its resources. The resource layer’s coupling association represents the relationships between resource elements, resulting from the need for resource sharing. This association arises in two ways: first, from the inherent correlation failure logic within the resource layer, reflecting the relationships between resources needed to perform system functions or tasks; second, once the shared resource configuration is finalized, associations between resource elements serving the same higher-level resource or function are established. After analyzing different resource configurations, we can determine the failure logical relationships for each scheme. These relationships can be inherent or generated by the specific resource configurations. To quantify the coupling relations in the resource layer, a specific definition or metric is required.

The association coefficient $\overset{n}{\underset{ij}{C}}$ is used to describe the association relationship between various resource elements, representing information exchange, material sharing, or resource contention between elements within the same layer after different resource configurations. The association information within the same resource layer can be represented by the association matrix $C^{n}$, which contains the association coefficients of shared resources at each layer:


$$\begin{array}{c} C^{n}={\left(\overset{n}{\underset{ij}{C}}\right)}_{m\times m}=\begin{array}{cc} \begin{array}{c} \begin{array}{c} \;\;\overset{n}{\underset{\text{1}}{r}} \end{array}\\ \begin{array}{c} \overset{n}{\underset{\text{2}}{r}}\\ \begin{array}{c} \vdots \\ \overset{n}{\underset{m}{r}} \end{array} \end{array} \end{array} & \begin{array}{c} \begin{array}{cc} \overset{n}{\underset{\text{1}}{r}} & \;\overset{n}{\underset{\text{2}}{r}} \end{array}\;\begin{array}{cc} \ldots & \overset{n}{\underset{m}{r}} \end{array}\\ \;[\begin{array}{ccc} \begin{array}{cc} \overset{n}{\underset{\text{1}\text{1}}{C}} & \overset{n}{\underset{\text{1}\text{2}}{C}}\\ \overset{n}{\underset{\text{2}\text{1}}{C}} & \overset{n}{\underset{\text{2}\text{2}}{C}} \end{array} & \cdots & \begin{array}{c} \overset{n}{\underset{\text{1}m}{C}}\\ \overset{n}{\underset{\text{2}m}{C}} \end{array}\\ \vdots & \ddots & \vdots \\ \begin{array}{cc} \overset{n}{\underset{m\text{1}}{C}} & \overset{n}{\underset{m\text{2}}{C}} \end{array} & \cdots & \overset{n}{\underset{mm}{C}} \end{array}] \end{array} \end{array} \end{array}$$ (9)

where $\overset{n}{\underset{ij}{C}}=\left\{ \begin{array}{c} \text{1},\text{ A correlation between resource }\overset{n}{\underset{i}{r}}\text{ and }\overset{n}{\underset{j}{r}}\;\\ \;\text{0},\text{ Not a correlation between resource }\overset{n}{\underset{i}{r}}\text{ and }\overset{n}{\underset{j}{r}} \end{array}\right.\;$ and $\overset{n}{\underset{mm}{C}}=\text{0}$. So the above formula can be expressed as:


$$\begin{array}{c} C^{n}={\left(\overset{n}{\underset{ij}{C}}\right)}_{m\times m}=\begin{array}{c} \begin{array}{cc} \begin{array}{c} \begin{array}{c} \overset{n}{\underset{\text{1}}{r}} \end{array}\\ \begin{array}{c} \overset{n}{\underset{\text{2}}{r}}\\ \begin{array}{c} \vdots \\ \overset{n}{\underset{m}{r}} \end{array} \end{array} \end{array} & \begin{array}{c} \;\;\;\;\;\begin{array}{cc} \overset{n}{\underset{\text{1}}{r}} & \;\overset{n}{\underset{\text{2}}{r}} \end{array}\;\begin{array}{cc} \;\ldots & \overset{n}{\underset{m}{r}} \end{array}\\ \left[\begin{array}{ccc} \begin{array}{cc} \text{0} & \overset{n}{\underset{\text{1}\text{2}}{C}}\\ \overset{n}{\underset{\text{2}\text{1}}{C}} & \text{0} \end{array} & \cdots & \begin{array}{c} \overset{n}{\underset{\text{1}m}{C}}\\ \overset{n}{\underset{\text{2}m}{C}} \end{array}\\ \vdots & \ddots & \vdots \\ \begin{array}{cc} \overset{n}{\underset{m\text{1}}{C}} & \overset{n}{\underset{m\text{2}}{C}} \end{array} & \cdots & \text{0} \end{array}\right] \end{array} \end{array} \end{array} \end{array}$$ (10)

Influence probability of failure propagation $\overset{n}{\underset{ij}{P}}$ is the failure probability of shared resources in the same resource layer affected by fault propagation. Failure association propagation probability matrix $C^{nP}$ of the same resource level is:


$$\begin{array}{c} C^{nP}={\left(\overset{nP}{\underset{ij}{C}}\right)}_{m\times m}=\begin{array}{cc} \begin{array}{c} \begin{array}{c} \;\\ \overset{n}{\underset{\text{1}}{r}} \end{array}\\ \begin{array}{c} \overset{n}{\underset{\text{2}}{r}}\\ \begin{array}{c} \vdots \\ \overset{n}{\underset{m}{r}} \end{array} \end{array} \end{array} & \begin{array}{c} \begin{array}{cc} \;\overset{n}{\underset{\text{1}}{r}} & \;\overset{n}{\underset{\text{2}}{r}}\; \end{array}\;\begin{array}{cc} \;\ldots & \;\overset{n}{\underset{m}{r}} \end{array}\\ \left[\begin{array}{ccc} \begin{array}{cc} \text{0} & \overset{n}{\underset{\text{1}\text{2}}{C}}\overset{n}{\underset{\text{1}\text{2}}{P}}\\ \overset{n}{\underset{\text{2}\text{1}}{C}}\overset{n}{\underset{\text{2}\text{1}}{P}} & \text{0} \end{array} & \cdots & \begin{array}{c} \overset{n}{\underset{\text{1}m}{C}}\overset{n}{\underset{\text{1}m}{P}}\\ \overset{n}{\underset{\text{2}m}{C}}\overset{n}{\underset{\text{2}m}{P}} \end{array}\\ \vdots & \ddots & \vdots \\ \begin{array}{cc} \overset{n}{\underset{m\text{1}}{C}}\overset{n}{\underset{m\text{1}}{P}} & \overset{n}{\underset{m\text{2}}{C}} \end{array}\overset{n}{\underset{m\text{2}}{P}} & \cdots & \text{0} \end{array}\right] \end{array} \end{array} \end{array}$$ (11)

The inherent failure probability of shared resource $\overset{n}{\underset{mb}{P}}$ is the inherent failure probability of the m shared resource in the n class resource.

The failure probability $\overset{n}{\underset{m}{P}}$ of each shared resource is not only related to the inherent failure probability $\overset{n}{\underset{mb}{P}}$ of the shared resource, but also affected by the failure propagation due to the existence of correlation, that is, the failure propagation influence probability $\overset{n}{\underset{ij}{P}}$. The expression of failure probability $\overset{n}{\underset{m}{P}}$ for each shared resource is:


$$\overset{n}{\underset{m}{P}}=\overset{n}{\underset{mb}{P}}+\sum_{i,j}\overset{n}{\underset{ij}{\overset{n}{\underset{ij}{C}}P}}$$ (12)

Particularly attention is: the shared resource set $\left(\overset{n}{\underset{\text{1}}{r}},\overset{n}{\underset{\text{2}}{r}},\overset{n}{\underset{\text{3}}{r}},\ldots ,\;\overset{n}{\underset{N_{m}}{r}}\right)$ at the next lower level allocate in the same resource $\overset{n+\text{1}}{\underset{m}{r}}$. If the upper resource $\overset{n+\text{1}}{\underset{m}{r}}$ fails, all the lower shared resources will fail under the influence of failure propagation.

The failure probability ${P(F}_{ij})$ represents the likelihood of the top event occurring in the failure logic relation of the IMA system. In summary, the failure probability is calculated by adding the probability of system failure due to upper-layer resource failure to that due to the upper-layer resource functioning correctly, considering both the inherent correlation logic of the complex system and the post-configuration shared resource correlations. The failure probability $\overset{n}{\underset{m}{P}}$ value for each shared resource can be deduced using formulas (2), (11), and (12).

2) Real-time performance

High real-time performance includes short response times to events and short task execution times. Task execution time is closely related to the allocation of time resources. Different allocations of time resources can result in varied system idle times, which in turn lead to different task execution times. Therefore, to represent the system’s real-time performance, we use the system idle time index, while ensuring that there is redundancy built into the system’s design schedule. The idle time of all tasks running on the *q*-th Integrated Processing Module (IPM) can be expressed as: $\overset{q}{\underset{idle\_time}{F}}\left(\overset{n}{\underset{m}{r}}\right)=\overset{q}{\underset{alloc}{Time}}-\overset{q}{\underset{used}{Time}}$, in which $\overset{q}{\underset{alloc}{Time}}$ is the total time allocated to the *q* th compute module, $\overset{q}{\underset{used}{Time}}$ is the total time consumed by all tasks. The total system idle time is then: $F_{idle\_time}=\sum_{q=\text{1}}^{Q}\overset{q}{\underset{idle\_time}{F}}\left(\overset{n}{\underset{m}{r}}\right)$. This metric reflects the schedulability margin and is used to evaluate real-time performance in our optimization.

### 4.2. Resource utilization efficiency model

The IMA system’s shared resource feature optimizes resource allocation, leading to better utilization efficiency. This study focuses on quantifying resource utilization efficiency to evaluate the effectiveness of resource sharing and isolation designs. Resource utilization efficiency is defined as the degree or quantity of shared resources used to achieve safe and accurate system operations during the configuration of resource sharing and isolation. It reflects whether resources are sharing or isolation and is a crucial indicator for measuring the extent of resource utilization.

The resource utilization efficiency of an IMA system is defined as follows:


$$UE(x,k)=\sum_{k=\text{1}}^{N}\frac{K_{r(k)}}{n_{used(k)}}$$ (13)

where, $K_{r(k)}$ represents the utilization efficiency influence factor of class k resources, k∈[1,2,…,x]. The higher the level of shared resources, the greater the utilization efficiency influence factor $K_{r(k)}$. $n_{used(k)}$ indicates the number of class k resources actually used, $n_{used(k)}=(\text{1},\text{2},\cdots ,N),N\geq N_{y}$. Note: every shared resource will be used, that is, $n_{used(k)}=\text{0}$ will not appear. The resource utilization efficiency is related to the number of shared resources used. The higher the degree of sharing, the lower the isolation degree, and the lower the number of shared resources used, the higher the resource utilization efficiency. Conversely, the lower the sharing degree is, the higher the isolation degree is, and the more shared resources are used, the lower the resource utilization efficiency.

${Count}_{x,y}$ counts the usage of the y shared resource object, $y\in \left[\text{1},N_{\text{y}}\right]$. Each configuration scheme can obtain a mapping association matrix, equation (2). Then, by calculating the elements in the n line of formula $M^{rf}$, we can get ${Count}_{x,y}$:


$${Count}_{x,y}=\sum_{j=\text{1}}^{m}\overset{rf}{\underset{nj}{M}}$$ (14)

in which, ${Count}_{x,y}\in [\text{1},m]$.

Then, $n_{used(k)}$ can be calculated by all elements in the formula $M^{rf}$:


$$n_{used(k)}=\sum {Count}_{x,y}=\sum_{i=\text{1},j=\text{1}}^{n,m}\overset{rf}{\underset{ij}{M}}$$ (15)

Generally, the optimal configuration scheme has the highest resource efficiency.

In addition, the configured number of shared resources $n_{used(k)}$ is related to the usage of the y shared resource object ${Count}_{x,y}$, $n_{used(k)}=N_{y}+\sum_{\text{1}}^{t}\overset{t}{\underset{x,y}{Count}}$. Assume that n resources need to be configured in m upper-layer resources and there are t type of shared resource objects. The value of $n_{used(k)}$ ranges from n to (n+t·m) shared resource objects. The constraint is described by an inequality:


$$n\leq \overset{rf}{\underset{ij}{A_{u}M}}A_{v}\leq n+t\cdot m$$ (16)

where, $A_{u}=[\text{1},\text{1},\cdots ,\text{1}]$, $A_{v}=[\text{1},\text{1},\cdots ,\text{1}{]}^{T}$. $n_{used(k)}=\overset{rf}{\underset{ij}{A_{u}M}}A_{v}=\sum_{i=\text{1},j=\text{1}}^{n,m}\overset{rf}{\underset{ij}{M}}$. 1≤t,1≤n,1≤m.

### 4.3. Multi-objective optimization of resource sharing and isolation design

To sum up, the constraints for shared resource configuration are presented in the following formulas. We combine these constraints with the objective function of the optimization to derive the standard multi-objective optimization model for shared resources:


$${P(F}_{ij}):\overset{n}{\underset{m}{P}},\;{P(F}_{ij})\leq P\left(F_{Req}\right)$$

$$\text{min}\;\{F_{idle_{time}}(\overset{\text{1}}{\underset{N\text{1}}{r}},\overset{\text{2}}{\underset{N\text{2}}{r}},\cdots ,\overset{n}{\underset{Nt}{r}})\}$$

$$\text{max}\;UE(x,k)=\sum_{k=\text{1}}^{N}\frac{K_{r(k)}}{n_{used(k)}}\;$$

$$s.t.\;\text{1}\leq k\leq n,\;b_{\text{1}}\leq M^{\text{1}\text{2}}A_{\text{1}}\leq b_{m}\;$$

$$\;{\;A}_{DR(rf)}\overset{rf}{\underset{nj}{M}}=b_{DR(rf)},\;A_{DR(fr)}\overset{rf}{\underset{im}{M}}\geq b_{DR(fr)}$$

$$A_{SEG}M^{rf}\leq b_{SEG},\;A_{SCR}\overset{rf^{\prime }}{\underset{im}{M}}=b_{SCR}$$

$$\;n\leq \overset{rf}{\underset{ij}{A_{u}M}}A_{v}\leq n+t\cdot m$$

            1≤t,1≤n,1≤m,n≤m (17)

The established multi-objective optimization model incorporates a variety of equality and inequality constraints. Typically, in the optimization process, the objective functions restrict each other, and the goal is to find an optimal solution that maximizes all target values. In recent years, extensive and in-depth research has been conducted on multi-objective optimization across various fields. This has led to the proposal of different multi-objective evolutionary algorithms. Researchers commonly employ two main categories of methods: mathematical programming and meta-heuristic approaches [27]. Although mathematical programming offers faster convergence and higher accuracy, it can be challenging to apply to more complex real-world systems. Consequently, meta-heuristic algorithms have become increasingly popular. Heuristic algorithms are considered the most effective for solving complex systems’ optimization problems related to reliability, safety, and economics [28].

The NSGA-II is a well-known multi-objective genetic algorithm that utilizes a non-dominated sorting approach, allowing for a satisfactory spread of solutions while achieving satisfactory convergence in the obtained non-dominated front [29]. NSGA-II integrates unique advantages, such as ranking, elitism, and the absence of shared parameters, and has demonstrated significant improvements in computing efficiency. This paper selected NSGA-II for the following reasons: 1) Pareto-Optimality Handling: NSGA-II efficiently generates a diverse set of Pareto-optimal solutions, which is essential for multi-objective optimization involving safety, efficiency, and real-time performance. 2) Non-Dominated Sorting: It ranks solutions based on dominance, allowing us to explore trade-offs between conflicting objectives without weighting. 3) Applicability to Constrained Problems: NSGA-II can handle the equality and inequality constraints defined in our model (Section 3.2) through constraint-domination principles. 4) Proven Effectiveness in Embedded Systems: NSGA-II has been widely adopted in avionics and embedded system optimization, demonstrating robustness and scalability for similar configuration problems. As a result, NSGA-II is used in this paper to address the multi-objective optimization problem of IMA resource sharing and isolation configuration.

## 5. Case study

In this section, we discuss the optimization process for configuring shared resources in a small-scale IMA system known as the Integrated Display and Control System (IDCS). The design of the IDCS is informed by empirical data and related research, as cited in references [30] and [31]. The IDCS is a complex modern integrated system with resource sharing as its main feature. The primary purpose of the IDCS is to present data from airborne sensors and systems to pilots and crew, enabling safe flight and mission completion. The IDCS consists of two main components: display and control. The display component serves as the input function for the pilot, while the control component serves as the output function.

The software of the Integrated Display Unit (IDU) is divided into two main parts: the application software and the platform software. These software components reside on the Input/Output Module (IOM), which serves as the General Processing Module (GPM) for the IDU. IDCS maps the logical and physical connections between software resource requirements and the platform’s hardware configuration. The platform-level tasks are broken down into essential functions, primarily display and control. The necessary resources for these functions are then effectively allocated to node, module, and partition resources.

**Fig 4:**
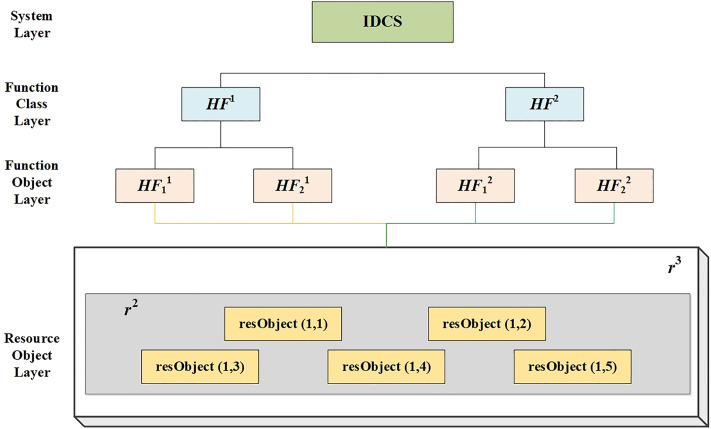
The function-resource implementation relationship structure of IDCS.

The IDCS case model constructed in this section is shown in Fig 4, the IDCS consists of three resource layers: node resources ($r^{\text{3}}$), module resources ($r^{\text{2}}$), and partition resources ($r^{\text{1}}$). Each sub-function is mapped to a set of resources, illustrating the sharing/isolation trade-off. $r^{\text{3}}$ represents node resources. $r^{\text{2}}$ stands for module resource, in this case the GPM is needed. $r^{\text{1}}$ represents a partition resource. A partition is the smallest unit of resource configuration and invocation in the system. A module can be configured into several partitions. Display function ${HF}^{\text{1}}$and control function ${HF}^{\text{2}}$ are each composed of two sub-functions, which are complementary to each other. It is important to note that in order to illustrate the effectiveness of the proposal approach, the IDCS examples in this article only cover these three types of resources.

**Fig 5:**
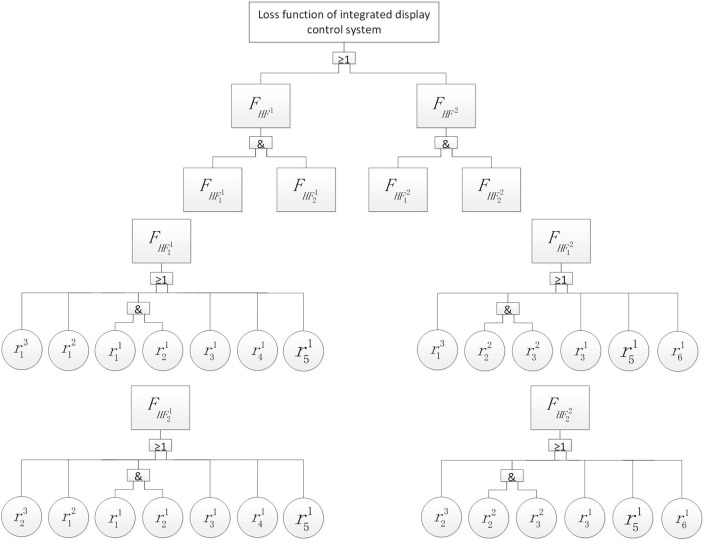
Inherent failure logic within IDCS.


In addition to its own failure, each shared resource is also affected by the failure propagation of its subordinate resources. Therefore, the inherent relationship between the function loss of this IDCS and resource failure can be divided into the following situations: When the third type of resource $r^{\text{3}}$ fails; When the third resource $r^{\text{3}}$ does not fail and the second resource $r^{\text{2}}$ fails; When the third resource $r^{\text{3}}$ and the second resource $r^{\text{2}}$ do not fail. To simplify the logical relationship of these three cases, there is a definite logic relationship in IDCS resource layer, and this inherent logic relationship (as shown in Fig 5) remains unchanged regardless of the amount and form of resource allocation. The two types of shared resources $r^{\text{3}}$ and $r^{\text{2}}$ involve sharing and isolation, the number of configurations is set to 2 and 3. Fig 5 depicts the inherent failure logic of IDCS resources. The diagram is used to derive the failure probability expressions in Eq. (18), independent of specific configurations. Therefore, the failure logic expression of the IDCS case is:

$$F_{IDCS}=\left(F_{\overset{\text{1}}{\underset{\text{1}}{HF}}}\cap F_{\overset{\text{1}}{\underset{\text{2}}{HF}}}\right)\cup \left(F_{\overset{\text{2}}{\underset{\text{1}}{HF}}}\cap F_{\overset{\text{2}}{\underset{\text{2}}{HF}}}\right)$$

$$F_{\overset{\text{1}}{\underset{\text{1}}{HF}}}=\text{F}(\overset{\text{3}}{\underset{\text{1}}{r}})\cup \text{F}(\overset{\text{2}}{\underset{\text{1}}{r}})\cup \left[\overset{\text{1}}{\underset{\text{1}}{F(r}})\cap \overset{\text{1}}{\underset{\text{2}}{F(r}})\right]\cup \overset{\text{1}}{\underset{\text{3}}{F(r}})\cup \overset{\text{1}}{\underset{\text{4}}{F(r}})\cup \overset{\text{1}}{\underset{\text{5}}{F(r}})$$

$$F_{\overset{\text{1}}{\underset{\text{2}}{HF}}}=\text{F}(\overset{\text{3}}{\underset{\text{2}}{r}})\cup \text{F}(\overset{\text{2}}{\underset{\text{1}}{r}})\cup \left[\overset{\text{1}}{\underset{\text{1}}{F(r}})\cap \overset{\text{1}}{\underset{\text{2}}{F(r}})\right]\cup \overset{\text{1}}{\underset{\text{3}}{F(r}})\cup \overset{\text{1}}{\underset{\text{4}}{F(r}})\cup \overset{\text{1}}{\underset{\text{5}}{F(r}})$$

$$F_{\overset{\text{2}}{\underset{\text{1}}{HF}}}=\text{F}(\overset{\text{3}}{\underset{\text{1}}{r}})\cup \left[\overset{\text{2}}{\underset{\text{2}}{F(r}})\cap \overset{\text{2}}{\underset{\text{3}}{F(r}})\right]\cup \overset{\text{1}}{\underset{\text{3}}{F(r}})\cup \overset{\text{1}}{\underset{\text{5}}{F(r}})\cup \overset{\text{1}}{\underset{\text{6}}{F(r}})$$

$$F_{\overset{\text{2}}{\underset{\text{2}}{HF}}}=\text{F}(\overset{\text{3}}{\underset{\text{2}}{r}})\cup \left[\overset{\text{2}}{\underset{\text{2}}{F(r}})\cap \overset{\text{2}}{\underset{\text{3}}{F(r}})\right]\cup \overset{\text{1}}{\underset{\text{3}}{F(r}})\cup \overset{\text{1}}{\underset{\text{5}}{F(r}})\cup \overset{\text{1}}{\underset{\text{6}}{F(r}})$$ (18)

### 5.1 IDCS safety quantization model

The key parameters used in the IDCS case study—including resource failure probabilities (Table 1), task parameters (Table 2), and NSGA-II settings—are provided in the Supporting Information dataset accompanying this paper.

**Table 1:** Inherent failure probability of various resource objects for IDCS.

Resource categories	Resource objects	Inherent failure probability
$r^{\text{3}}$	$\overset{\text{3}}{\underset{\text{1}}{r}}$	1 × 10^−8^
	$\overset{\text{3}}{\underset{\text{2}}{r}}$	1 × 10^−8^
$r^{\text{2}}$	$\overset{\text{2}}{\underset{\text{1}}{r}}$	1 × 10^−7^
	$\overset{\text{2}}{\underset{\text{2}}{r}}$	1 × 10^−7^
	$\overset{\text{2}}{\underset{\text{3}}{r}}$	1 × 10^−7^
$r^{\text{1}}$	$\overset{\text{1}}{\underset{\text{1}}{r}}$	3 × 10^−5^
	$\overset{\text{1}}{\underset{\text{2}}{r}}$	3 × 10^−5^
	$\overset{\text{1}}{\underset{\text{3}}{r}}$	1 × 10^−5^
	$\overset{\text{1}}{\underset{\text{4}}{r}}$	1 × 10^−6^
	$\overset{\text{1}}{\underset{\text{5}}{r}}$	1 × 10^−6^
	$\overset{\text{1}}{\underset{\text{6}}{r}}$	1 × 10^−5^

**Table 2:** Task parameter.

Task	Computing resource consumption (time resource consumption)	Interface resource consumption	Required peripheral
Task1	10(3)	0	0
Task2	1(0.5)	9	2 type 1 peripherals
Task3	0(0)	5	1 type 2 peripherals
Task4	10(3)	0	0
Task5	1(0.5)	9	1 type 1 peripherals
Task6	0(0)	5	2 type 2 peripherals
Task7	20(5)	0	0
Task8	0(0)	6	1 type 3 peripherals
Task9	20(5)	0	0
Task10	0(0)	6	1 type 3 peripherals

In this model of case, the number of resources that have realized functions is $N_{\text{1}}=\text{6}$、$N_{\text{2}}=\text{3},N_{\text{3}}=\text{2}$. Table 1 shows the resource objects and inherent failure probability of various resources in the IDCS system.

In this case, the probability of failure propagation is given as $\overset{\text{1}}{\underset{ij}{P}}={\text{1}\text{0}}^{-\text{6}}$，$\overset{\text{2}}{\underset{ij}{P}}={\text{1}\text{0}}^{-\text{8}}$.

In this case, there are three types of shared resources: $r^{\text{3}}$、$r^{\text{2}}$、$\overset{\text{1}}{\underset{\text{1}}{r}}$、$\overset{\text{1}}{\underset{\text{2}}{r}}$、$\overset{\text{1}}{\underset{\text{3}}{r}}$、$\overset{\text{1}}{\underset{\text{4}}{r}}$、$\overset{\text{1}}{\underset{\text{5}}{r}}$、$\overset{\text{1}}{\underset{\text{6}}{r}}$. Among them, $\overset{\text{3}}{\underset{\text{1}}{r}}$、$\overset{\text{3}}{\underset{\text{2}}{r}}$、$\overset{\text{2}}{\underset{\text{1}}{r}}$、$\overset{\text{2}}{\underset{\text{2}}{r}}$、$\overset{\text{2}}{\underset{\text{3}}{r}}$ are designed for sharing and isolation. $\overset{\text{1}}{\underset{\text{1}}{r}}$、$\overset{\text{1}}{\underset{\text{2}}{r}}$、$\overset{\text{1}}{\underset{\text{4}}{r}}$、$\overset{\text{1}}{\underset{\text{6}}{r}}$ are designed to be shared without isolation, and $\overset{\text{1}}{\underset{\text{1}}{r}}$, $\overset{\text{1}}{\underset{\text{2}}{r}}$ are conflicting resources. For shared resource $\overset{\text{1}}{\underset{\text{3}}{r}}$, the number of configurations is 1–2: When it is adopted sharing design, number of configurations is 1; And when it is adopted isolated design, number of configurations is 2, and for the sub-functions $\overset{\text{1}}{\underset{\text{1}}{HF}}$ and $\overset{\text{2}}{\underset{\text{1}}{HF}}$ use resource $\overset{\text{1}}{\underset{\text{3}(\text{1})}{r}}$ and $\overset{\text{1}}{\underset{\text{3}(\text{2})}{r}}$. The shared resource $\overset{\text{1}}{\underset{\text{5}}{r}}$ is a dedicated resource, so the number of this resources is configured as 4, that is, each shared resource in the second type of resource $r^{\text{2}}$ corresponds to one dedicated resource $\overset{\text{1}}{\underset{\text{5}}{r}}$, then the sub-functions for $\overset{\text{1}}{\underset{\text{1}}{HF}}$ and $\overset{\text{2}}{\underset{\text{1}}{HF}}$ use $\overset{\text{1}}{\underset{\text{5}(\text{1})}{\text{r}}}$. At this time, the sub-function $\overset{\text{1}}{\underset{\text{1}}{HF}}$ uses$\overset{\text{1}}{\underset{\text{5}(\text{2})}{\text{r}}}$, and the sub-function $\overset{\text{2}}{\underset{\text{1}}{HF}}$ uses $\overset{\text{1}}{\underset{\text{5}(\text{3})}{r}}$; Shared resource $\overset{\text{1}}{\underset{\text{6}}{r}}$ is a safety-critical resource.

In addition, this case has given the allocation results of the second type of shared resources $\overset{\text{2}}{\underset{\text{1}}{r}}$, $\overset{\text{2}}{\underset{\text{2}}{r}}$, $\overset{\text{2}}{\underset{\text{3}}{r}}$ allocated in $\overset{\text{3}}{\underset{\text{1}}{r}}$, $\overset{\text{3}}{\underset{\text{2}}{r}}$, that is, $\overset{\text{2}}{\underset{\text{1}}{r}}$, $\overset{\text{2}}{\underset{\text{2}}{r}}$ allocated in $\overset{\text{3}}{\underset{\text{1}}{r}}$, and $\overset{\text{2}}{\underset{\text{3}}{r}}$ allocated in $\overset{\text{3}}{\underset{\text{2}}{r}}$. So the association matrix of the second type resources and propagation probability of failure influence have been determined.

In summary, according to the inherent failure logic of IDCS system, the failure probability of integrated display control system can be calculated as follows:


$${P_{\left(IDCS\right)}(F}_{ij})=\text{1}-\left[\text{1}-P\left(F_{\overset{\text{1}}{\underset{\text{1}}{HF}}}\right)\cdot P\left(F_{\overset{\text{1}}{\underset{\text{2}}{HF}}}\right)\right]\cdot \left[\text{1}-P\left(F_{\overset{\text{2}}{\underset{\text{1}}{HF}}}\right)\cdot P\left(F_{\overset{\text{2}}{\underset{\text{2}}{HF}}}\right)\right]$$ (19)

In this case, there are 10 tasks, including 6 computing tasks and 6 interface tasks (two of which are both computing tasks and interface tasks). Computing tasks are allocated to GPM module, while interface tasks are allocated to Remote Data Collector (RDC) module. Different computing tasks consume different time resources. Different IPM devices may lead to different idle time. Table 2 lists the resources and peripherals required for each task.

According to the description of the construction objective function in Section 4, system idle time $F_{idle\_time}=\sum_{\text{6}}\overset{q}{\underset{idle\_time}{F}}$ can be obtained corresponding to this case. In which, $\overset{q}{\underset{idle\_time}{F}}=\overset{q}{\underset{Nm}{R}}-\overset{q}{\underset{task}{R}}$. $\overset{q}{\underset{Nm}{R}}$ indicates the time allocated by the *q* th resource. $\overset{q}{\underset{task}{R}}$ represents the time resource consumed by all tasks running on the *q* th module.

### 5.2 IDCS resource utilization efficiency model

Resource utilization efficiency measures the extent to which resources are utilized. According to the calculation of system resource utilization efficiency formula (15), the resource utilization efficiency of this IDCS case is:


$${UE}_{\left(IDCS\right)}(x,k)=\frac{K_{r(\text{1})}}{n_{used(\text{1})}}+\frac{K_{r(\text{2})}}{n_{used(\text{2})}}+\frac{K_{r(\text{3})}}{n_{used(\text{3})}}$$ (20)

The impact factor of IDCS case resources utilization efficiency is given as: $K_{r(\text{1})}=\text{1}/\text{1}\text{2}\text{5}$, $K_{r(\text{2})}=\text{1}/\text{5}$, $K_{r(\text{3})}=\text{1}/\text{2}$. $\overset{\text{3}}{\underset{\text{1}}{r}}$, $\overset{\text{3}}{\underset{\text{2}}{r}}$, $\overset{\text{2}}{\underset{\text{1}}{r}}$, $\overset{\text{2}}{\underset{\text{2}}{r}}$, $\overset{\text{2}}{\underset{\text{3}}{r}}$ are shared and isolated design, and the number of configurations is given. $\overset{\text{1}}{\underset{\text{1}}{r}}$, $\overset{\text{1}}{\underset{\text{2}}{r}}$, $\overset{\text{1}}{\underset{\text{4}}{r}}$, and $\overset{\text{1}}{\underset{\text{6}}{r}}$ are configured as shared resources without isolation design. Therefore, ① When the shared resource $\overset{\text{1}}{\underset{\text{3}}{r}}$ adopts the shared design, the number of configurations is 1. ② When the shared resource $\overset{\text{1}}{\underset{\text{3}}{r}}$ is isolated, the number of shared resources is 2. Table 3 shows the actual number of IDCS resource objects.

**Table 3:** The actual used number of IDCS resource objects.

Situation	Resource objects	Actual used number
$r^{\text{3}}$	$r^{\text{3}}$	2
	$r^{\text{2}}$	3
	$r^{\text{1}}$	8
$r^{\text{2}}$	$r^{\text{3}}$	2
	$r^{\text{2}}$	3
	$r^{\text{1}}$	9

### 5.3 Safety constraints modeling

The IMA system is subject to many different safety constraints during the configuration of shared resources. The specific safety constraints of this IDCS case are as follows:

1) The bottom shared resources are all used

In this IDCS case, there are 6 kinds of bottom-layer shared resources, including $\overset{\text{1}}{\underset{\text{1}}{r}}$, $\overset{\text{1}}{\underset{\text{2}}{r}}$, $\overset{\text{1}}{\underset{\text{3}}{r}}$, $\overset{\text{1}}{\underset{\text{4}}{r}}$, $\overset{\text{1}}{\underset{\text{5}}{r}}$, and $\overset{\text{1}}{\underset{\text{6}}{r}}$, which are all configured. The mathematical description that meets this safety constraint is as follows:


$$b_{\text{1}(IDCS)}\leq M^{\text{1}\text{2}}A_{\left(IDCS\right)}\leq b_{\text{3}(IDCS)}$$ (21)

among them, $A_{\left(IDCS\right)}=[\text{1},\text{1},\text{1},\text{1},\text{1},\text{1}{]}^{T}$,$b_{\text{1}(IDCS)}=[\text{1},\text{1},\text{1}{]}^{T},\;b_{\text{3}(IDCS)}=[\text{3},\text{3},\text{3}{]}^{T}$.

2) Constrain on the number of dedicated resources

As shown in the previous Section 4, shared resource $\overset{\text{1}}{\underset{\text{5}}{r}}$ is a dedicated resource and cannot be shared. It supports only one upper-layer resource $r^{\text{2}}$, that is, each upper-layer resource $r^{\text{2}}$ corresponds to one dedicated resource $\overset{\text{1}}{\underset{\text{5}}{r}}$. Thus, there are three dedicated resources: $\overset{\text{1}}{\underset{\text{5}(\text{1})}{r}}$, $\overset{\text{1}}{\underset{\text{5}(\text{2})}{r}}$, $\overset{\text{1}}{\underset{\text{5}(\text{3})}{r}}$, which allocate respectively in the upper resources $\overset{\text{2}}{\underset{\text{1}}{r}}$, $\overset{\text{2}}{\underset{\text{2}}{r}}$, $\overset{\text{2}}{\underset{\text{3}}{r}}$, $\sum_{j=\text{1}}^{\text{3}}\overset{rf}{\underset{\text{5}j}{M}}=\text{1}$. Describe this safety constraint with a mathematical formula:


$$A_{DR(IDCS)}\overset{rf}{\underset{\text{5}j}{M}}=b_{DR(IDCS)}$$ (22)

In this case, $A_{DR(IDCS)}=[\text{1},\text{1},\text{1}{]}^{T}$, $b_{DR(IDCS)}=[\text{1},\text{1},\text{1}{]}^{T}$.

In addition, these three dedicated resources are configured in upper layer resource $r^{\text{2}}$ like other lower-layer shared resources. Therefore, the number of resources in the upper layer resource allocated by dedicated resources is greater than or equal to 1, $\sum_{i=\text{1}}^{n}\overset{rf\text{1}}{\underset{im}{M}}\geq \text{1}$. The constraint is expressed by the inequality as:


$$A_{DR(fr)}\overset{rf\text{1}}{\underset{im}{M}}\geq b_{DR(fr)}$$ (23)

where $A_{DR(fr)}=[\text{1},\text{1},\cdots ,\text{1}]$, $b_{DR(fr)}=[\text{1},\text{1},\cdots ,\text{1}]$. In this case, the association configuration matrix $\overset{rf\text{1}}{\underset{im}{M}}$ is different from $\overset{rf}{\underset{ij}{M}}$. It is equivalent to the associated configuration matrix when dedicated resources $\overset{\text{1}}{\underset{\text{5}(\text{1})}{r}}$, $\overset{\text{1}}{\underset{\text{5}(\text{2})}{r}}$, $\overset{\text{1}}{\underset{\text{5}(\text{3})}{r}}$ are simultaneously configured with other five shared resources $\overset{\text{1}}{\underset{\text{1}}{r}}$, $\overset{\text{1}}{\underset{\text{2}}{r}}$, $\overset{\text{1}}{\underset{\text{3}}{r}}$, $\overset{\text{1}}{\underset{\text{4}}{r}}$, $\overset{\text{1}}{\underset{\text{6}}{r}}$.

3) Conflicting resources cannot be configured in the same upper-layer resource, and isolation constraints must be met

In the IDCS case, there are conflicting resources: the shared resources $\overset{\text{1}}{\underset{\text{1}}{r}}$ and $\overset{\text{1}}{\underset{\text{2}}{r}}$ are conflicting resources, so they cannot be configured in the same upper layer resource $\overset{\text{2}}{\underset{N_{\text{2}}}{r}}$. Therefore, the correlation configuration matrix: $\overset{\overset{\text{1}}{\underset{\text{1}}{r}}\overset{\text{2}}{\underset{N_{\text{2}}}{r}}}{\underset{\text{1}m}{M}}+\overset{\overset{\text{1}}{\underset{\text{2}}{r}}\overset{\text{2}}{\underset{N_{\text{2}}}{r}}}{\underset{\text{2}m}{M}}\leq \text{1}$. The constraint is described by an inequality:


$$A_{SEG(IDCS\text{1})}M^{rf}\leq b_{SEG(IDCS\text{1})}$$ (24)

in which, $A_{SEG(IDCS\text{1})}=\left[\begin{array}{ccc} \text{1} & \text{1} & \text{0}\\ \text{1} & \text{1} & \text{0} \end{array}\;\begin{array}{ccc} \text{0} & \text{0} & \text{0}\\ \text{0} & \text{0} & \text{0} \end{array}\right],$ $b_{SEG(IDCS\text{1})}=[\begin{array}{ccc} \text{1} & \text{1} & \text{1}\\ \text{1} & \text{1} & \text{1} \end{array}]$.

In addition, when the shared resource $\overset{\text{1}}{\underset{\text{3}}{r}}$ adopts the isolation design, its configuration quantity is 2, namely $\overset{\text{1}}{\underset{\text{3}(\text{1})}{r}}$ and $\overset{\text{1}}{\underset{\text{3}(\text{2})}{r}}$. At this time, the two shared resources $\overset{\text{1}}{\underset{\text{3}(\text{1})}{r}}$ and $\overset{\text{1}}{\underset{\text{3}(\text{2})}{r}}$ are also conflicting resources, so they cannot be configured in the same upper layer resource $\overset{\text{2}}{\underset{N_{\text{2}}}{r}}$. Namely $\overset{\overset{\text{1}}{\underset{\text{3}(\text{1})}{r}}\overset{\text{2}}{\underset{N_{\text{2}}}{r}}}{\underset{\text{3}(\text{1})m}{M}}+\overset{\overset{\text{1}}{\underset{\text{3}(\text{2})}{r}}\overset{\text{2}}{\underset{N_{\text{2}}}{r}}}{\underset{\text{3}(\text{2})m}{M}}\leq \text{1}$. The constraint is described by an inequality:


$$A_{SEG(IDCS\text{2})}M^{rf^{\text{2}}}\leq b_{SEG(IDCS\text{2})}$$ (25)

where, $A_{SEG(IDCS\text{2})}=\left[\begin{array}{ccc} \text{0} & \text{0} & \text{1}\\ \text{0} & \text{0} & \text{1} \end{array}\;\begin{array}{ccc} \text{0} & \text{0} & \text{0}\\ \text{0} & \text{0} & \text{0} \end{array}\;\begin{array}{ccc} \text{0} & \text{0} & \text{1}\\ \text{0} & \text{0} & \text{1} \end{array}\right]$, $b_{SEG(IDCS\text{2})}=[\begin{array}{ccc} \text{1} & \text{1} & \text{1}\\ \text{1} & \text{1} & \text{1} \end{array}]$. In this case, the associated configuration matrix $M^{rf^{\text{2}}}$ is different from that of $M^{rf}$. It is equivalent to the associated configuration matrix of the nine resources $\overset{\text{1}}{\underset{\text{1}}{r}}$, $\overset{\text{1}}{\underset{\text{2}}{r}}$, $\overset{\text{1}}{\underset{\text{3}(\text{1})}{r}}$, $\overset{\text{1}}{\underset{\text{5}(\text{1})}{r}}$, $\overset{\text{1}}{\underset{\text{4}}{r}}$, $\overset{\text{1}}{\underset{\text{6}}{r}}$, $\overset{\text{1}}{\underset{\text{5}(\text{2})}{r}}$, $\overset{\text{1}}{\underset{\text{5}(\text{3})}{r}}$, and $\overset{\text{1}}{\underset{\text{3}(\text{2})}{r}}$.

4) Safety-critical resources must be isolated

In this case, shared resource $\overset{\text{1}}{\underset{\text{6}}{r}}$ is a safety critical resource and must be isolated. That is, there is only one shared resource $\overset{\text{1}}{\underset{\text{6}}{r}}$ and one dedicated resource $\overset{\text{1}}{\underset{\text{5}(g)}{r}}$ in the upper-layer resource $\overset{\text{2}}{\underset{N_{\text{2}}}{r}}$ allocated to it. Therefore, the two value of the distribution of correlation matrix: $\left\{\overset{\overset{\text{1}}{\underset{\text{6}}{r}}\overset{\text{2}}{\underset{\text{1}}{r}}}{\underset{\text{1}\text{6}}{M}},\overset{\overset{\text{1}}{\underset{\text{6}}{r}}\overset{\text{2}}{\underset{\text{2}}{r}}}{\underset{\text{2}\text{6}}{M}},\overset{\overset{\text{1}}{\underset{\text{6}}{r}}\overset{\text{2}}{\underset{\text{3}}{r}}}{\underset{\text{3}\text{6}}{M}}\right\}$ have and only have 1, namely the $\sum_{i=\text{1}}^{\text{3}}\overset{\overset{\text{1}}{\underset{\text{6}}{r}}r^{\text{2}}}{\underset{i\text{6}}{M}}=\text{2}$. Use an equality expression to describe this constraint:


$$A_{SCR(IDCS)}\overset{\overset{\text{1}}{\underset{\text{6}}{r}}r^{\text{2}}}{\underset{i\text{6}}{M}}=b_{SCR(IDCS)}$$ (26)

in which, $A_{SCR(IDCS)}=[\text{1},\text{1},\cdots ,\text{1}]$, $b_{SCR(IDCS)}=[\text{2},\text{2},\text{2}]$.

This case contains t=8 configurations of shared resource objects, but there is one safety critical resource and two conflicted resources, reducing the number of shared resources, the actual number of these three configurations is 1. In addition, there is one dedicated resource, so the actual shared resource constraint configured in this case is $\text{14}\leq n_{used(IDCS)}\leq \text{22}$. In order to better illustrate the proposed method, only the sharing and isolation design of $\overset{\text{1}}{\underset{\text{3}}{r}}$ shared resource is analyzed, and the sharing and isolation form of other shared resources has been designed. Table Ⅱ shows the usage of shared resources.

According to the civil aircraft airworthiness regulation CCAR25.1309 [31,32], the quantitative index of aircraft failure should be less than 10^−9^. The failure of this kind of system will prevent continued safe flight and landing and cause a catastrophic accident. Therefore, in this IDCS case, the failure probability is required to be $P\left(F_{Req}\right)={\text{1}\text{0}}^{-\text{9}}$.

In summary, the optimization model of this IDCS case is shown as follow:


$${P_{\left(IDCS\right)}(F}_{ij})=\text{1}-\left[\text{1}-P\left(F_{\overset{\text{1}}{\underset{\text{1}}{HF}}}\right)\right]\cdot \left[\text{1}-P\left(F_{\overset{\text{1}}{\underset{\text{2}}{HF}}}\right)\right]$$

$$minF_{idle\_time}=\sum_{\text{6}}\overset{q}{\underset{idle\_time}{F}}$$

$$max\;{UE}_{\left(IDCS\right)}(x,k)=\frac{K_{r(\text{1})}}{n_{used(\text{1})}}+\frac{K_{r(\text{2})}}{n_{used(\text{2})}}+\frac{K_{r(\text{3})}}{n_{used(\text{3})}}$$

$${P_{\left(IDCS\right)}(F}_{ij})\leq {\text{1}\text{0}}^{-\text{9}}$$

s.t.1≤k≤3

$$b_{\text{1}(IDCS)}\leq M^{\text{1}\text{2}}A_{\left(IDCS\right)}\leq b_{\text{3}(IDCS)}$$

$$A_{\left(IDCS\right)}=[\text{1},\text{1},\text{1},\text{1},\text{1},\text{1}{]}^{T},b_{\text{1}(IDCS)}=[\text{1},\text{1},\text{1}{]}^{T},b_{\text{3}(IDCS)}=[\text{3},\text{3},\text{3}{]}^{T}$$

$$A_{DR(IDCS)}\overset{rf}{\underset{\text{5}j}{M}}=b_{DR(IDCS)}$$

$$A_{DR(IDCS)}=[\text{1},\text{1},\text{1}{]}^{T},b_{DR(IDCS)}=[\text{1},\text{1},\text{1}{]}^{T}$$

$$A_{DR(fr)}\overset{rf\text{1}}{\underset{im}{M}}\geq b_{DR(fr)}$$

$$A_{DR(fr)}=[\text{1},\text{1},\cdots ,\text{1}],b_{DR(fr)}=[\text{1},\text{1},\cdots ,\text{1}]$$

$$A_{SEG(IDCS\text{1})}M^{rf}\leq b_{SEG(IDCS\text{1})}$$

$$\begin{array}{c} A_{SEG(IDCS\text{1})}=\left[\begin{array}{ccc} \text{1} & \text{1} & \text{0}\\ \text{1} & \text{1} & \text{0} \end{array}\;\begin{array}{ccc} \text{0} & \text{0} & \text{0}\\ \text{0} & \text{0} & \text{0} \end{array}\right],b_{SEG(IDCS\text{1})}=[\begin{array}{ccc} \text{1} & \text{1} & \text{1}\\ \text{1} & \text{1} & \text{1} \end{array}] \end{array}$$

$$A_{SEG(IDCS\text{2})}M^{rf^{\text{2}}}\leq b_{SEG(IDCS\text{2})}$$

$$\begin{array}{c} A_{SEG(IDCS\text{2})}=\left[\begin{array}{ccc} \text{0} & \text{0} & \text{1}\\ \text{0} & \text{0} & \text{1} \end{array}\;\begin{array}{ccc} \text{0} & \text{0} & \text{0}\\ \text{0} & \text{0} & \text{0} \end{array}\;\begin{array}{ccc} \text{0} & \text{0} & \text{1}\\ \text{0} & \text{0} & \text{1} \end{array}\right],\;b_{SEG(IDCS\text{2})}=[\begin{array}{ccc} \text{1} & \text{1} & \text{1}\\ \text{1} & \text{1} & \text{1} \end{array}] \end{array}$$

$$A_{SCR(IDCS)}\overset{\overset{\text{1}}{\underset{\text{6}}{r}}r^{\text{2}}}{\underset{i\text{6}}{M}}=b_{SCR(IDCS)}$$

$$A_{SCR(IDCS)}=[\text{1},\text{1},\cdots ,\text{1}],b_{SCR(IDCS)}=[\text{2},\text{2},\text{2}]$$

$$\text{14}\leq n_{used(IDCS)}\leq \text{22}$$ (27)

### 5.4 Configuration Optimization

**Fig 6:**
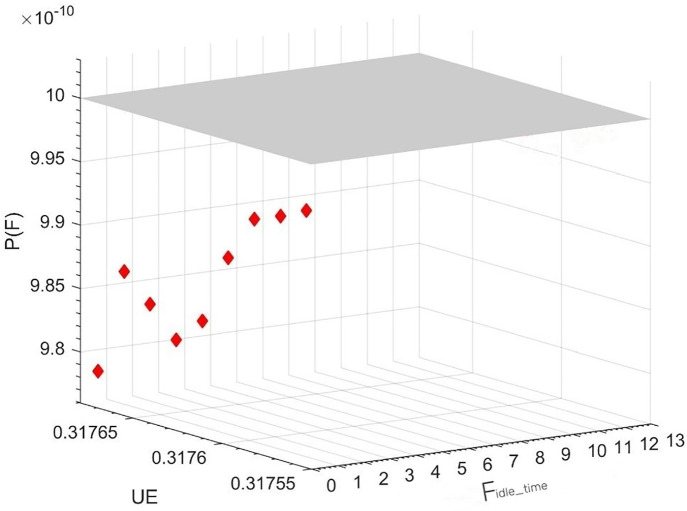
Pareto bound solution space for IDCS system resource configuration optimization.

Based on the conditions and preliminary analysis of the IDCS case, the optimization model for shared resource allocation in the integrated display control system can be solved using the NSGA-II optimization algorithm, as discussed in Section 3.3. For this example, the NSGA-II algorithm parameters are set as follows: population quantity $n_{p}=\text{20}$; maximum genetic algebra $G_{max}=\text{100}$; genetic crossover ratio $P_{c}=\text{0}.\text{8}$; proportion of genetic variation $P_{m}=\text{0}.\text{3}$; mutation probability μ=0.7. The final Pareto solution set is obtained through iterative evolution, and its corresponding state position is shown in Fig 6.

**Fig 7:**
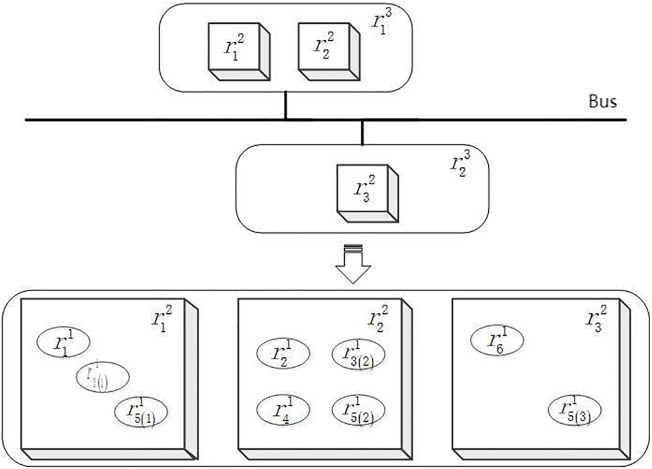
The optimal configuration scheme of IDCS.

The NSGA-II-based configuration scheme optimization algorithm generates a relatively large number of Pareto solution sets, from which a configuration scheme that aligns with the optimization objective can be selected. This paper aims to achieve optimal efficiency while maintaining system safety. As a result, 9 groups of Pareto solutions that satisfy these requirements have been identified in this example. These solutions meet the safety requirements and have the same optimal resource utilization efficiency ${UE}_{\left(IDCS\right)}(x,k)=\text{0}.\text{31767}$. To identify the optimal solution, the one with the lowest failure probability and shortest idle time is selected, and the optimal configuration scheme of this IDCS case is presented in Fig 7.

**Fig 8:**
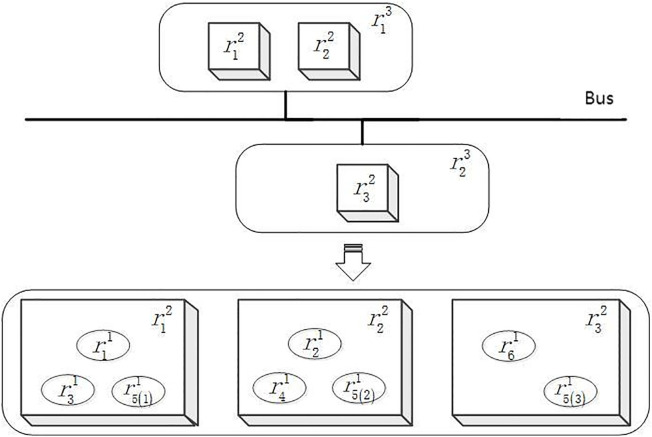
Configuration scheme example of $\overset{1}{\underset{3}{𝐫}}$ shared design.

The configuration scheme depicted in Fig 4 shows the shared resource $\overset{\text{1}}{\underset{\text{3}}{r}}$ that is adopted in isolation design, with a configuration quantity of 1. To better illustrate the difference between sharing and isolation configurations, a configuration scheme was selected from the Pareto solution set when $\overset{\text{1}}{\underset{\text{3}}{r}}$ is adopted in shared design, as shown in Fig 8. This scheme was compared and analyzed with the optimal scheme depicted in Fig 7. The comparison of their optimization target values is presented in Table 4. It is evident from the two configuration schemes that although the shared resource $\overset{\text{1}}{\underset{\text{3}}{r}}$ in Fig 8 utilizes the shared design with higher resource utilization efficiency, resource sharing exacerbates the risk of fault propagation, which does not meet the safety requirements of the IDCS system. Therefore, while the shared design can enhance the system’s efficiency, it also increases the risk, while the isolation design can enhance the system’s safety and reduce the probability of fault propagation.

**Table 4:** Comparison with the optimal configuration scheme.

	$\overset{1}{\underset{3}{𝐫}}$ isolation design	$\overset{1}{\underset{3}{𝐫}}$ sharing design
${P_{\left(IDCS\right)}(F}_{ij})$	$\text{0}.\text{978869}{\times \text{1}\text{0}}^{-\text{9}}$	$\text{1}.\text{00142}{\times \text{1}\text{0}}^{-\text{9}}$
${UE}_{\left(IDCS\right)}(x,k)$	0.31767	0.31756
$F_{idle\_time}$	4	6

To sum up, the configuration of each resource in the optimal configuration scheme of this IDCS case is shown in Table 5.

**Table 5:** Resource configuration of sub-functions.

Sub-function	Resource configuration
$\overset{\text{1}}{\underset{\text{1}}{HF}}$	$\overset{\text{3}}{\underset{\text{1}}{r}}$、$\overset{\text{2}}{\underset{\text{1}}{r}}$、$\overset{\text{1}}{\underset{\text{1}}{r}}$、$\overset{\text{1}}{\underset{\text{2}}{r}}$、$\overset{\text{1}}{\underset{\text{3}}{r}}$、$\overset{\text{1}}{\underset{\text{4}}{r}}$、$\overset{\text{1}}{\underset{\text{5}(\text{1})}{r}}$
$\overset{\text{2}}{\underset{\text{1}}{HF}}$	$\overset{\text{3}}{\underset{\text{2}}{r}}$、$\overset{\text{2}}{\underset{\text{1}}{r}}$、$\overset{\text{1}}{\underset{\text{1}}{r}}$、$\overset{\text{1}}{\underset{\text{2}}{r}}$、$\overset{\text{1}}{\underset{\text{3}}{r}}$、$\overset{\text{1}}{\underset{\text{4}}{r}}$、$\overset{\text{1}}{\underset{\text{5}(\text{2})}{r}}$
$\overset{\text{2}}{\underset{\text{1}}{HF}}$	$\overset{\text{3}}{\underset{\text{1}}{r}}$、$\overset{\text{2}}{\underset{\text{2}}{r}}$、$\overset{\text{2}}{\underset{\text{3}}{r}}$、$\overset{\text{1}}{\underset{\text{3}}{r}}$、$\overset{\text{1}}{\underset{\text{5}(\text{1})}{r}}$、$\overset{\text{1}}{\underset{\text{6}}{r}}$
$\overset{\text{2}}{\underset{\text{1}}{HF}}$	$\overset{\text{3}}{\underset{\text{2}}{r}}$、$\overset{\text{2}}{\underset{\text{2}}{r}}$、$\overset{\text{2}}{\underset{\text{3}}{r}}$、$\overset{\text{1}}{\underset{\text{3}}{r}}$、$\overset{\text{1}}{\underset{\text{5}(\text{3})}{r}}$、$\overset{\text{1}}{\underset{\text{6}}{r}}$

## 6. Conclusion

To ensure safety and prevent hazards and resource conflicts associated with shared resources, it is critical to establish a reasonable configuration scheme during system development. This scheme should efficiently manage resource sharing and isolation. Different configurations can lead to varying levels of risk related to fault propagation and coupling faults, highlighting the need to balance resource sharing and isolation.

This paper presents a method for optimizing the shared resource configuration scheme in the IMA system, focusing on modes for resource sharing and isolation. Safety and utilization efficiency are formalized as constraints in the configuration scheme evaluation, with mathematical expressions for these constraints to enhance computational capabilities.

The main contributions of this paper include: 1) establishing a configuration optimization model for multi-objective resource sharing and isolation design; 2) providing mathematical descriptions for safety constraints in resource configuration; 3) developing an optimization analysis method for resource sharing and isolation from safety and efficiency perspectives, offering technical support for efficient and safe resource configuration optimization in IMA systems.

While the proposed method demonstrates promising results, this research has several limitations. Currently, it employs only the failure probability model and real-time performance as quantitative safety indicators. Future studies should consider incorporating other critical safety indicators, such as fault tolerance, diagnosability, and system modifiability, to provide a more comprehensive safety assessment. Additionally, the optimization model assumes static configurations and relies on parameters such as failure probabilities, which may be uncertain in early design phases. Scalability to large-scale IMA systems with hundreds of resources requires further investigation, possibly through heuristic pruning or parallelization. Moreover, practical deployment faces challenges such as certification compliance (e.g., DO-297, ARINC 653) and integration with existing model-based engineering toolchains.

Future work will focus on: (1) extending the model to support dynamic reconfiguration for adaptive fault tolerance; (2) integrating the optimizer with system-level safety analysis tools (e.g., STPA, FTA) to automate constraint derivation; (3) developing decision-support interfaces to help designers navigate Pareto-optimal solutions; (4) incorporating broader safety metrics such as fault containment and modifiability; and (5) validating the approach on larger, industrial-scale IMA platforms in collaboration with avionics partners.These directions aim to enhance the method’s applicability, robustness, and integration into real-world avionics development processes.

## Supporting information

S1 Dataset IDCS resource/task data and NSGA-II algorithm parameters. (XLSX)
